# Cerebral Blood Flow Difference Between Acute and Chronic Tinnitus Perception: A Perfusion Functional Magnetic Resonance Imaging Study

**DOI:** 10.3389/fnins.2021.752419

**Published:** 2021-10-05

**Authors:** Jinghua Hu, Jin-Jing Xu, Song’an Shang, Huiyou Chen, Xindao Yin, Jianwei Qi, Yuanqing Wu

**Affiliations:** ^1^Department of Otolaryngology, Nanjing First Hospital, Nanjing Medical University, Nanjing, China; ^2^Department of Radiology, Nanjing First Hospital, Nanjing Medical University, Nanjing, China

**Keywords:** cerebral blood flow, tinnitus perception, magnetic resonance imaging, arterial spin labeling, perfusion

## Abstract

**Purpose:** The central nervous mechanism of acute tinnitus is different from that of chronic tinnitus, which may be related to the difference of cerebral blood flow (CBF) perfusion in certain regions. To verify this conjecture, we used arterial spin labeling (ASL) perfusion magnetic resonance imaging (MRI) in this study to compare the CBF alterations of patients with acute and chronic tinnitus.

**Methods:** The current study included patients with chronic tinnitus (*n* = 35), acute tinnitus (*n* = 30), and healthy controls (*n* = 40) who were age-, sex-, and education-matched. All participants underwent MRI scanning and then ASL images were obtained to measure CBF of the entire brain and analyze the differences between groups as well as the correlations with tinnitus characteristics.

**Results:** The chronic tinnitus group showed increased z-CBF in the right superior temporal gyrus (STG) and superior frontal gyrus (SFG) when compared with the acute tinnitus patients. Further connectivity analysis found enhanced CBF connectivity between the right STG and fusiform gyrus (FG), the right SFG and left middle occipital gyrus (MOG), as well as the right parahippocampal gyrus (PHG). Moreover, in the chronic tinnitus group, the tinnitus handicap questionnaire (THQ) score was positively correlated with the normalized z-CBF of right STG (*r* = 0.440, *p* = 0.013).

**Conclusion:** Our results confirmed that the CBF changes in some brain regions were different between acute and chronic tinnitus patients, which was correlated with certain tinnitus characteristics. This is of great value to further research on chronicity of tinnitus, and ASL has a promising application in the measurement of CBF.

## Introduction

Tinnitus is the perception of an auditory sensation without a corresponding external sound stimulus (Biswas et al., 2019). It is a global problem with a prevalence of about 10%–15% in adults worldwide (Henry et al., 2020). A unified classification system for tinnitus has not been realized. In the “A multidisciplinary European guideline for tinnitus: diagnostics, assessment, and treatment” published in 2019, tinnitus is divided into acute tinnitus (duration ≤ 3 months), subacute tinnitus (duration < 3 months), and chronic tinnitus (duration ≥ 6 months) (Cima et al., 2019). Clinical assessments of tinnitus including objective and subjective assessments showed different clinical features between acute and chronic tinnitus, such as tinnitus loudness, frequency, and tinnitus-related mood disorders (Wallhäusser-Franke et al., 2017). A recent longitudinal study found that 18.4% of acute tinnitus patients had complete remission of tinnitus within 6 months. There was no change in tinnitus characteristics in patients with their tinnitus persistent (Vielsmeier et al., 2020). However, the exact mechanism of the chronization of tinnitus remains a mystery.

In recent years, there have been many neuroimaging techniques used to study the central nervous mechanism of tinnitus like electroencephalography (EEG), magnetoencephalography (MEG), and functional magnetic resonance imaging (fMRI) (Nathan et al., 2005; Han et al., 2018; Berlot et al., 2020; Chen et al., 2021). It seems that the auditory system may play a basic role in tinnitus generation (Møller, 2007). Meanwhile, some non-auditory areas, such as prefrontal, parietal, and limbic regions, are closely related to tinnitus maintenance, severity, the accompanying emotional disorder, and cognitive impairment (Chen et al., 2017b, 2018; Zimmerman et al., 2019). However, most studies have analyzed chronic or acute tinnitus independently. The specific differences of neural mechanism between the acute and chronic tinnitus as well as the mechanism of transformation from acute to chronic tinnitus are rarely investigated. Vanneste et al. (2011) detected enhanced neural activity in the right auditory cortex in chronic tinnitus compared with recent-onset tinnitus using the EEG approach. A latest EEG study compared acute and chronic tinnitus, which found differences in neural activity and connectivity in many regions. It was found that a non-auditory brain region, especially parahippocampus gyrus, plays a key role in the transition from acute to chronic tinnitus (Lan et al., 2021). Therefore, we suggest that chronization of tinnitus is a complex mechanism involving multiple networks.

Besides structural and functional brain alterations, positron emission tomography (PET) and single photo emission computed tomography (SPECT) studies confirmed abnormal cerebral blood flow (CBF) of patients with tinnitus (Lanting et al., 2009; Ueyama et al., 2015). It was found that regional CBF in tinnitus patients was lower in DMN regions and higher in memory and emotional networks (Ueyama et al., 2015). Reyes et al. (2002) used SPECT and found that changes in tinnitus loudness can cause significant CBF changes in the auditory cortex of the temporal lobe. So, the abnormal CBF in tinnitus may be related to the tinnitus sound stimulations, but we do not know if it has anything to do with the duration of the stimulus.

Arterial spin labeling (ASL) is another technique to investigate brain hemodynamic changes. Different from PET and SPECT, ASL is a completely non-invasive method without using invasive radioactive tracers (Ferré et al., 2013). Moreover, ASL could provide reproducible and reliable quantitative measurements of cerebral perfusion non-invasively (Telischak et al., 2015). In terms of quantitative measurement of CBF, ASL is more advantageous than blood oxygen level-dependent MRI (BOLD-MRI) (Detre and Wang, 2002; Kim et al., 2019). ASL has been used to quantify CBF alterations in many diseases, such as Parkinson’s disease (Barzgari et al., 2019) and Alzheimer’s disease (Zhang et al., 2017). Recently, a few researches used ASL to study CBF alterations in tinnitus patients (Li X. et al., 2020; Li et al., 2021). However, the objects of these studies were pulsatile tinnitus patients, which is different from subjective tinnitus in symptoms, etiology, mechanism, and so on (Pegge et al., 2017). Some recent studies have explored the regional CBF alterations in subjective tinnitus patients with other complications, such as diabetes (Xia et al., 2020) or migraine (Xu et al., 2021). However, no studies have evaluated the role of CBF in tinnitus chronization mechanisms so far. The regional CBF can be used to reflect the neural activity in local brain regions. When different brain regions cooperate to complete a certain function, regional CBF may change synchronously (Li F. et al., 2020). Therefore, CBF connectivity is also an important indicator, which is similar to the structural and functional connectivity.

Based on the above, the current study used ASL technology to calculate and compare CBF changes of acute and chronic tinnitus patients. We hypothesized that different CBF patterns may exist in different stages of tinnitus chronization. A further study on the mechanism of tinnitus chronicity may be of great help to provide a new imaging perspective for early diagnosis and prognosis evaluation.

## Materials and Methods

This study was approved by the Research Ethics Committee of the Nanjing Medical University. All the subjects signed written informed consent before participating in the study.

### Participants

From May 2018 to January 2020, we recruited 30 acute tinnitus patients (duration < 1 month, 11.0 ± 6.9 days) and 35 chronic tinnitus patients (duration > 6 months, 46.1 ± 39.4 months) from the Department of Otolaryngology and 40 healthy examinees as healthy controls (HC) in Nanjing First Hospital of Nanjing Medical University. All subjects were 20–70 years old, right-handed, and completed at least 8 years of education. All tinnitus patients presented bilateral subjective tinnitus without obvious causes. None of them had received any form of tinnitus treatment (including medication, acupuncture, and electrical stimulation) before entering the study.

**FIGURE 1:**
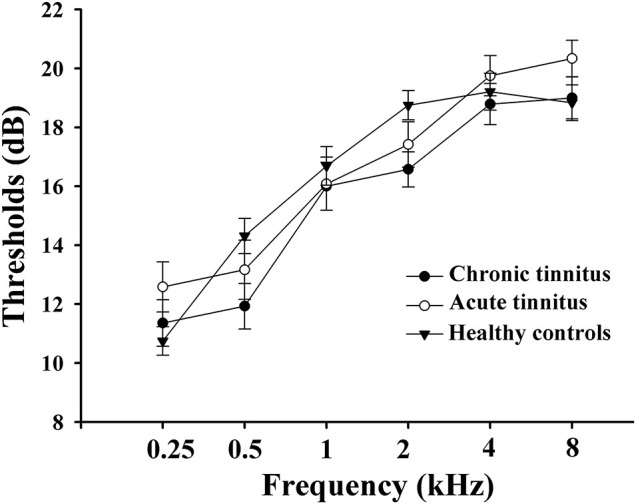
Mean hearing thresholds of the chronic tinnitus, acute tinnitus, and healthy controls. Data are presented as mean ± SEM.

All subjects were tested by puretone audiometry (PTA) to determine the hearing thresholds at the frequencies of 0.25, 0.5, 1, 2, 4, and 8 kHz. Normal hearing means hearing thresholds < 25 dB and mild hearing loss means hearing thresholds between 26 and 40 dB (Olaosun and Ogundiran, 2013). All HC and most tinnitus patients had normal hearing. Only a few cases of tinnitus patients had mild sensorineural hearing loss. There were no significant differences among acute tinnitus patients, chronic tinnitus patients, and HC in auditory thresholds (Figure 1).

All the patients filled in the following questionnaires: (1) the Iowa version Tinnitus handicap questionnaire (THQ) (Kuk et al., 1990) to assess the severity of tinnitus; (2) the Self-Rating Depression Scale (SDS) for self-rated depression status; (3) the Self-Rating Anxiety Scale (SAS) for self-rated anxiety status; and (4) the Mini-Mental State Examination (MMSE) to assess the cognitive function. A MMSE score of < 26 indicates cognitive impairment, which is one of the exclusion criteria. None of the participants in this study had depression, anxiety, and mild cognitive impairment according to these tests above.

Participants were also excluded in the current study if they have the following conditions: (1) hyperacusis [detected by the Hyperacusis Questionnaire (Khalfa et al., 2002)]; (2) objective tinnitus, pulsatile tinnitus, Meniere’s diseases, history of use of ototoxic drugs, or ear surgery and other major ear diseases; (3) bad habits that may affect the nervous system (e.g., severe smoking, alcoholism, and drug addiction), neurological or psychiatric illness such as serious insomnia, brain injury, schizophrenia, and Alzheimer’s disease, Parkinson’s disease, and peripheral neuropathy; (4) major medical illness such as cancer, blood system diseases, and thyroid dysfunction; and (5) contraindications for MRI such as heart pacemakers, artificial heart valves, aneurysm clips, metal objects in the body, and claustrophobia. The characteristics of the acute tinnitus and chronic tinnitus patients and HC are shown in Table 1.

**TABLE 1:** Demographic and clinical characteristics of all subjects.

	Chronic tinnitus (*n* = 35)	Acute tinnitus (*n* = 30)	Healthy controls (*n* = 40)	*p* value
Age (year)	51.11±11.38	49.73±12.17	45.10±11.04	0.064^a^
Education (year)	12.26±2.84	12.80±3.68	12.73±2.51	0.719^a^
Gender (male/female)	10/25	14/16	15/25	0.322^b^
SAS	40.80±5.60	37.93±6.16	38.08±5.72	0.073^a^
SDS	41.46±6.21	40.50±5.48	41.03±4.54	0.777^a^
THQ score PTA of left ear (dB HL) PTA of right ear (dB HL) Mean PTA of both ears (dB HL)	53.24±13.94 15.26±2.86 16.50±2.64 15.61±2.05	47.45±15.27 16.22±2.91 16.89±3.48 16.56±2.80	– 16.71±2.77 16.65±2.41 16.68±1.80	0.115^c^ 0.089^a^ 0.857^a^ 0.086^a^

*Data are expressed as Mean ± SD.*

*^a^The p values are obtained by using one-way analysis of variance.*

*^b^The p values are obtained by using χ2 test.*

*^c^The p values are obtained by using a two-sample t test.*

*SAS, Self-Rating Anxiety Scale; SDS, Self-Rating Depression Scale; THQ, tinnitus handicap questionnaire; PTA, puretone audiometry.*

### Magnetic Resonance Imaging Measurements

During the MRI scan, the subjects kept their eyes closed, stayed awake and remained still, and avoided thinking about anything in particular. We used foam padding to reduce the head motion, and none of subjects in this study was excluded because of excessive head motion (with over 2.0 mm translation or 2.0° rotation in any direction). We also used earplugs (Hearos Ultimate Softness Series, United States) to reduce the perception of the scanner noise, and they could attenuate the noise by approximately 32 dB, according to the manufacturer’s data.

Imaging was performed on a 3.0-T MRI scanner (Ingenia, Philips Medical Systems, Netherlands) with an 8-channel receiver array head coil. Imaging parameters are as follows: (1) A three-dimensional turbo fast echo (3D-TFE) T1-weighted imaging (T1WI) sequence with high resolution for structural images: acquisition matrix = 256 × 256, field of view (FOV) = 256 × 256 mm^2^, repetition time (TR)/echo time (TE) = 8.1/3.7 ms, flip angle (FA) = 8°, slices = 170, thickness = 1 mm, gap = 0 mm. The structural sequence scanning time totaled 5 min and 29 s. (2) A 3D-pseudocontinuous arterial spin labeling (pCASL) sequence for ASL-CBF images: TR = 4,000 ms; TE = 11 ms; post-label delay = 2,000 ms; FA = 90°; slice thickness = 4 mm with 10% gap; FOV = 240 × 240 mm^2^; matrix = 64 × 64; 24 axial slices, label duration = 1,650 ms. The ASL sequence scanning time totaled 4 min and 8 s.

### Data Processing and Cerebral Blood Flow Analysis

We used ASL data processing toolbox (ASLtbx) (Wang et al., 2008) and statistical parameter mapping software 12 (SPM12) to process MRI data. The pCASL data were processed to generate CBF map and quantitative CBF was calculated on this basis (Xu et al., 2010). The images were rearranged and adjusted to correct head movement. After non-linear transformation, the CBF images of 40 HC subjects were co-registered with the PET template in the Montreal Neurological Institute (MNI) space. The average co-registered CBF images of the HC came to be the MNI-standard CBF template. Each CBF image of acute and chronic tinnitus patients was co-registered to this MNI-standard CBF template (voxel size was 1.5 mm × 1.5 mm × 1.5 mm) and then was spatially smoothed with a Gaussian kernel [width at half maximum (FWHM) is 8 mm]. Normalization CBF map was obtained by dividing the CBF per voxel by the average CBF across the entire brain (Aslan and Lu, 2010).

### Gray Matter Volume Calculation

The gray matter volume (GMV) calculation was performed by using SPM12. The structural images were tissue classified in a standard uniform segmentation model and the cerebral tissues can be segmented into gray matter (GM), white matter (WM), and cerebrospinal fluid. The GM concentration map was initial affine registered to the MNI space (resampled to the voxel size of 1.5 mm × 1.5 mm × 1.5 mm). Then, a non-linear deformation of GM concentration image was carried out. Multiplying the non-linear determinant and GM concentration together results in the GMV of each voxel. Then, all the GMVs were smoothed using a Gaussian kernel of FWHM 10 mm. After spatial pre-processing of the data, the voxel-wised GMV maps were used in the later ASL analysis as covariates.

### Cerebral Blood Flow Connectivity Analysis

According to previous studies (Andersson et al., 2000; Xia et al., 2020), the clusters with significant group differences in CBF of tinnitus patients were selected as seed regions of interest (ROIs) including parieto-temporal auditory cortex, frontal paralimbic areas, and posterior cingulate cortex. To determine whether the ROIs have abnormal CBF connectivity, a multiple regression model was used to detect the CBF connectivity between each ROI seed and other voxels in the whole brain for each group. Age, gender, and GMV were included in this model as confounding covariates. The three groups were compared in pairs, and the CBF connectivity maps of each pair were combined to form a spatial mask to which the CBF of each voxel of each ROI was then correlated. For each pair of voxels, the slopes of CBF correlation reflect the difference in CBF connectivity between groups. A two-sample *t*-test was established within the spatial mask of the CBF connectivity map of ROI to analyze the differences in CBF connectivity between each ROI and all the other voxels in the brain so that we obtain a voxel map that shows significantly different CBF connectivity among acute tinnitus patients, chronic tinnitus patients, and HC subjects for each ROI. Multiple comparisons were corrected using the false discovery rate (FDR) correction (significance set at *p* < 0.01).

### Statistical Analyses

Statistical analyses were performed using the SPSS software (version 20.0; SPSS, Chicago, IL, United States). One-way analysis of variance (ANOVA) was used to compare the differences in demographic information and clinical measures among three groups. A *post hoc* test was used for comparison between the tinnitus and HC groups (*t*-test for means and χ^2^ test for proportions). Significance level was set at *p* < 0.05. For normalized CBF, the discrete sequence was Z transformed and the result was expressed as z-CBF. To extract regions that were significantly different in z-CBF between groups, a VBM analysis and two-sample *t*-test were used to perform group comparisons between the tinnitus and HC group. For CBF connectivity, a two-sample *t*-test was performed to extract areas with significantly different CBF connectivity between two groups. The results were corrected by age, gender, education level, and GMV and represented in the MNI coordinate system. The statistical value of the *t*-test was expressed as *T*-value and was corrected by FDR correction (significance threshold was set as *p* < 0.01).

In order to compute the correlations between the tinnitus characteristics and abnormal CBF as well as CBF connectivity, Pearson’s correlation based on ROI analysis was performed (age, gender, education level, and GMV as the correction factors). *p* < 0.05 was set as the significance threshold.

## Results

### Normalized Cerebral Blood Flow Differences Between Groups

**FIGURE 2:**
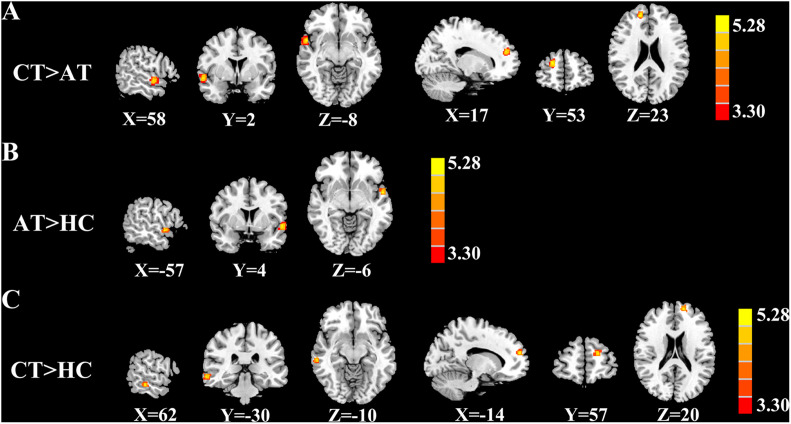
Significant CBF differences among the chronic tinnitus (CT), acute tinnitus (AT), and healthy controls (HC). **(A)** Compared to AT, CT showed increased z-CBF in the right superior temporal gyrus (STG), and right superior frontal gyrus (SFG). **(B)** Compared to HC, AT showed higher z-CBF in the left STG. **(C)** Compared to HC, CT showed increased z-CBF in right middle temporal gyrus (MTG) and left SFG. Significant thresholds were corrected using FDR criterion and set at *p* < 0.01.

Figure 2 and Table 2 show brain regions with different normalized CBF between groups. It turned out that z-CBF increased in the right superior temporal gyrus (STG) and superior frontal gyrus (SFG) of chronic tinnitus patients, when compared with the acute tinnitus subjects. Increased z-CBF in the right middle temporal gyrus (MTG) and left SFG was observed in the chronic tinnitus group compared to that in the HC group. Furthermore, acute tinnitus patients showed higher z-CBF in the left STG compared to the HC group. Moreover, no significant differences of GM and WM volumes among chronic tinnitus, acute tinnitus patients, and healthy controls were observed.

**TABLE 2:** Brain regions with significant group differences in normalized CBF with GMV correction.

Brain regions	BA	Peak MNI coordinates x, y, z (mm)	Peak *T* value	Cluster size (voxels)
**CT>AT**				
R_STG	22	58, 2, −8	4.405	163
R_SFG	210	17, 53, 23	5.588	455
**AT>HC**				
L_STG	22	−57, 4, −6	4.984	231
**CT>HC**				
R_MTG	21	62, −30, −10	4.488	325
L_SFG	10	−14, 57, 20	4.499	142

*Thresholds were set at a corrected *p*< 0.01 corrected by FDR criterion. MNI, Montreal Neurological Institute; CT, chronic tinnitus; AT, acute tinnitus; HC, healthy control; STG, superior temporal gyrus; SFG, superior frontal gyrus; MTG, middle temporal gyrus; L, left; R, right.*

### Cerebral Blood Flow Connectivity Differences Between Groups

**FIGURE 3:**
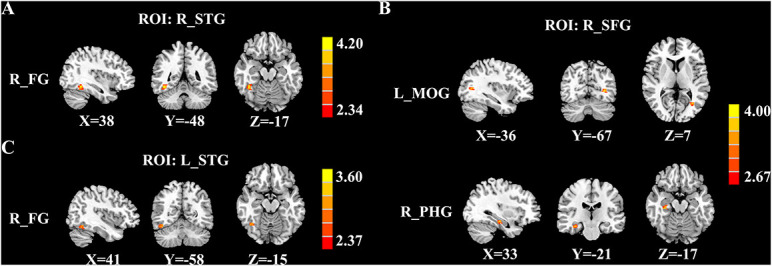
Significant group differences in CBF connectivity among the chronic tinnitus (CT), acute tinnitus (AT), and healthy controls (HC). **(A)** Compared with AT patients, CT patients exhibited increased CBF connectivity between the right superior temporal gyrus (STG) and the right fusiform gyrus (FG). **(B)** Compared with AT patients, CT patients also showed increased CBF connectivity between the right superior frontal gyrus (SFG) and the left middle occipital gyrus (MOG) as well as the right parahippocampal gyrus (PHG). **(C)** Compared with HC, the CT patients showed increased CBF connectivity between the left STG and the right FG. Significant thresholds were corrected using FDR criterion and set at *p* < 0.01.

Figure 3 and Table 3 depicted the group differences on CBF connectivity. When making a comparison with acute tinnitus, the chronic tinnitus group exhibited enhanced CBF connectivity between the seed ROI of the right STG and fusiform gyrus (FG). Moreover, it was observed in chronic tinnitus patients that the CBF connectivity aggrandized between the seed ROI of the right SFG and left middle occipital gyrus (MOG) as well as right parahippocampal gyrus (PHG). CBF connections between the seed ROI of the left STG and right FG were increased in chronic tinnitus patients compared to the HC group. There were no significant differences in CBF connectivity in seed of the right MTG and left SFG between groups.

**TABLE 3:** Brain regions with significant group differences in CBF connectivity.

ROI	Brain regions	BA	Peak MNI coordinates x, y, z (mm)	Peak *T* value	Voxels
R_STG	R_FG	37	38, −48, −17	4.276	157
R_SFG	L_MOG	19	−36, −67, 7	3.738	172
	R_PHG	36	33, −21, −17	3.571	107
L_STG	R_FG	37	41, −58, −15	3.475	112

*Thresholds were set at a corrected *p*<0.01 corrected by FDR criterion. ROI, region of interest; MNI, Montreal Neurological Institute; STG, superior temporal gyrus; FG, fusiform gyrus; SFG, superior frontal gyrus; MOG, middle occipital gyrus; PHG, parahippocampal gyrus; L, left; R, right.*

### Relationship Between z- Cerebral Blood Flow and Clinical Characteristics

**FIGURE 4:**
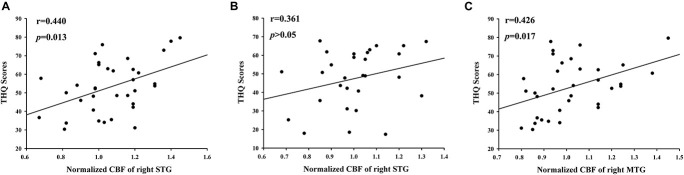
The significant correlations between the CBF changes and the tinnitus characteristics. The THQ score was positively correlated with the normalized z-CBF of the right STG in chronic tinnitus patients (*r* = 0.440, *p* = 0.013) **(A)**, but not in acute tinnitus patients (*r* = 0.361, *p* > 0.05) **(B)**. Compared with HC, the THQ score was positively correlated with the normalized z-CBF of the right MTG in chronic tinnitus patients (*r* = 0.426, *p* = 0.017) **(C)**.

Figure 4 illustrates the correlations between the z-CBF alteration and the tinnitus characteristics. Compared with acute tinnitus, the THQ score was significantly positively related with the z-CBF of the right STG in chronic tinnitus patients (*r* = 0.440, *p* = 0.013) but not in acute tinnitus patients (*r* = 0.361, *p* > 0.05). Besides, compared with HC, the THQ score was positively correlated with the z-CBF of the right MTG in chronic tinnitus patients (*r* = 0.426, *p* = 0.017). Finally, the z-CBF and CBF connectivity in other brain regions was not significantly correlated with specific tinnitus characteristics.

## Discussion

In this study, ASL-MRI was firstly used to compare the CBF perfusion differences between acute and chronic tinnitus. In comparison to the acute tinnitus group, the chronic tinnitus patients showed different CBF mainly in the STG, SFG, and MTG. Further ROI analysis exhibited that CBF connectivity was enhanced between the right STG and FG, right SFG and PHG, as well as the left MOG. In addition, in the chronic tinnitus group, there were positive correlations between THQ score and increased CBF in ROIs of temporal gyrus.

### The Temporal Gyrus

Compared to the acute tinnitus group, chronic tinnitus patients had increased z-CBF in the right STG. As part of the auditory cortex, the STG is critical for high-order auditory processing of speech by encoding phonetic features (Yi et al., 2019). It is believed that tinnitus is caused by an abnormal GM structure and neural activity of the central auditory cortex (Aldhafeeri et al., 2012; Chen et al., 2015a,c; Cai et al., 2019). Several researchers have also found increased CBF in the temporal auditory cortex of tinnitus patients using PET and SPECT (Andersson et al., 2000; Ueyama et al., 2015). However, the correlation between this change and tinnitus remains to be proved. Similar to our results, in the EEG study of Vanneste et al., increased neural electrical activity was found in the right auditory cortex in chronic tinnitus relative to recent-onset tinnitus (Vanneste et al., 2011). According to the tinnitus model proposed by Eggermont and Roberts (2004), the initial signal of ringing in the ears is due to abnormal nerve activity of auditory nerve fibers caused by peripheral auditory pathway injury. The abnormal neural activity causes other auditory neuron hyperactivity, which has a similar edge frequency. If the abnormity persists, the auditory cortex will reorganize in response to the change, mimicking the response to normal acoustic stimuli. That is why chronic tinnitus can lead to central alterations (Eggermont, 2003). It was found that in tinnitus patients in the resting state without external sound stimulation, the α activity of neurons in the temporal cortex was reduced. The longer the duration of tinnitus, the less variability of this α activity (Schlee et al., 2014). This reflects a reduced adaptation of the auditory cortex to persistent tinnitus. Therefore, we hypothesize that long-term abnormal auditory stimuli lead to changes in the auditory cortex, which are different from those in the acute phase, including an increase of CBF.

Moreover, the right FG and left MOG were found to have enhanced CBF connectivity to the right STG and SFG of chronic tinnitus brain in comparison to acute tinnitus. The FG is located in the ventral temporal cortex. FG and the visual area of the MOG belong to the visual center, which participates in advanced visual processing (Weiner and Zilles, 2016). Both visual and auditory processing engage the attention networks. Burton et al. (2012) found that in patients with annoying tinnitus, the functional activity of the visual cortex, including the occipital lobe and the temporoparietal junction, was negatively correlated with that of the auditory cortex. It means that when the blood oxygen activity increased in the auditory area, the activity of the visual area decreased, and vice versa. This may reflect an adaptation mechanism of the tinnitus brain: to focus attention on non-auditory tasks to reduce the salience of tinnitus sound. We hypothesized that during the acute phase of tinnitus, the brain is in a compensatory state and can make these adaptations above. However, when tinnitus signals exist for a long time and take up too much attention processing resources, tinnitus patients need to mobilize more attention during visual processing. So, a decompensated change happens and the attention network is widely active. This may explain our results showing increased CBF connectivity between the auditory-visual attention networks in patients with chronic tinnitus. Husain et al. (2015) compared central differences in processing auditory and visual attention in chronic tinnitus patients. They found that when tinnitus patients were in the visual modality, attention and short-term memory networks were more responsive, suggesting that tinnitus sufferers are more likely to be distracted than normal people when processing visual signals and thus become aware of their tinnitus (Husain et al., 2015). These results suggest that using attention demanding tasks to divert attention away from tinnitus may help reduce the severity of tinnitus (Searchfield et al., 2007).

In addition, the functional connectivity between STG and limbic area, cerebellum, and thalamus was found to be abnormal in chronic tinnitus, which was correlated with tinnitus-related characteristics including tinnitus-related emotional disorders, tinnitus severity, and tinnitus duration (Zhang et al., 2015; Chen et al., 2017b; Feng et al., 2018). These results demonstrated the important role of the auditory cortex in tinnitus. Therefore, we suggest that changes in CBF and CBF connectivity in the auditory region of the temporal gyrus may be involved in the chronicity of tinnitus. We also observed different CBF values in bilateral temporal gyrus of tinnitus brain, with enhanced CBF in the right STG, while no significant difference in the left STG. There is no clear evidence of a link between this asymmetry and tinnitus characteristics so far. However, some scholars believe that the hemisphere asymmetry of activity in the auditory cortex is a general characteristic of the normal brain unrelated to tinnitus brain (Geven et al., 2014).

### The Frontal Gyrus

Increased z-CBF in the right SFG was observed in the chronic tinnitus group compared to the acute tinnitus group. The SFG is a vital component of the auditory connection cortex, which takes part in processing multi-sensory signals, including auditory perception. Therefore, SFG may also be involved in the perception of tinnitus (Melloni et al., 2007; Chen et al., 2014). According to Chen et al., the bilateral SFG presented stronger network centrality, which suggested that the prefrontal cortex, especially SFG, is the major cortical hub of the tinnitus model (Chen et al., 2016). What is more, they found that tinnitus duration was significantly correlated with the increased amplitude of low-frequency fluctuation (ALFF) in SFG. ALFF is an indicator to reflect the intention of neural activity when the brain is in resting state. This result suggested that SFG could play a specific role in chronicity of tinnitus (Chen et al., 2015b).

The prefrontal part of the SFG is one of the ingredients of the default mode network (DMN), which was proved to be responsible for memory, emotion, and intrinsic control networks (Raichle, 2015). Decreased functional connectivity (FC) within the DMN may be vulnerable to chronic tinnitus patients with cognitive impairment (Chen et al., 2018). Schmidt et al. (2017) revealed decreased correlations between the DMN and the precuneus in long-term tinnitus when compared to recent-onset tinnitus (who had tinnitus for >6 months but <1 year) so that DMN–precuneus decoupling can be responsible for tinnitus persistent perception and a potential marker of chronic tinnitus (Schmidt et al., 2017).

Prefrontal cortex (PFC) also plays a basic role in the frontostriatal circuit, which is a top-down gating system involved in the brain “reward” mechanism (McGinty and Grace, 2009). This circuit is also associated with cognitive functions including regulation of attention tasks and learning. For example, the striatum is responsible for rapid “stimulus response” to the learning content, while the PFC is responsible for memory and storage (Antzoulatos and Miller, 2011). Thompson and Neugebauer (2019) suggested that this system plays an important role in the central nervous mechanism of chronic pain. It is believed that both chronic pain and tinnitus are sensory disorders. They are highly similar in their central mechanisms, among which the frontostriatal circuit may be the key (Rauschecker et al., 2015; Xu et al., 2019).

### The Limbic System

Our study showed enhanced CBF connectivity between the right SFG and PHG in chronic tinnitus patients. PHG is one of the key structures of the limbic system. Previous studies have shown abnormal changes in the structure, neural activity, and CBF of the PHG in tinnitus patients (Laureano et al., 2014; Chen et al., 2017a; Schmidt et al., 2018). Actually, the auditory and memory/limbic networks are closely interconnected in the perception of sound (Ćurčić-Blake et al., 2017). In a tinnitus model proposed by Rauschecker et al., the initial tinnitus was caused by the impairment of the auditory pathway, and the limbic system can prevent tinnitus signals from reaching the auditory cortex, thereby eliminating tinnitus (Rauschecker et al., 2010). Dysfunction of the limbic system affects this elimination mechanism, leading to persistent perception of tinnitus.

Meanwhile, the PHG plays a central role in memory recollection by sending information from the hippocampus to the related areas (Diederen et al., 2010). De Ridder et al. (2006) suggested that tinnitus memory was constantly updated due to the abnormal continuous activity of the PHG, which contributed to the dysfunction of tinnitus adaptation mechanism and finally led to the maintenance of tinnitus. Lan et al. (2021) found that the abnormal brain regions of acute tinnitus patients were mainly concentrated in the auditory cortex, while chronic tinnitus involved a larger brain network, in which the PHG showed significantly enhanced connectivity. This is similar to our result. All these results highlight that the PHG may be a very vital region to distinguish acute tinnitus from chronic tinnitus.

In addition to memory function, abnormalities in the structure and function of the PHG may be associated with the development of emotion disorders (Almeida et al., 2009). Jastreboff (1990) proposed that whether tinnitus patients develop negative emotions such as depression and anxiety depends on whether the limbic system is involved in central changes. Depression and anxiety, in turn, modulate the structural effects of tinnitus brain and further aggravate tinnitus by enhancing the detection and perception of tinnitus through specific patterns (Besteher et al., 2019). The PHG also plays an important role in the formation and maintenance of bound information (Luck et al., 2010). In this way, tinnitus patients are more likely to bundle some uncomfortable symptoms with tinnitus, which further increases the anxiety and fear of tinnitus. This is obviously more conducive to the elimination of tinnitus. Although the interaction mode between tinnitus and negative emotions is not completely clear, it is certain that the long-term perception of tinnitus is related to the memory and emotion mechanism mediated by the limbic system.

### Cerebral Blood Flow Connectivity

Cerebral blood flow connectivity changes in tinnitus patients are firstly reported in the current study. CBF of different brain regions is not independent, which can reflect the changes of neuronal activity. Synchronous changes of CBF connectivity may occur in areas of the same functional network (Havsteen et al., 2018; Jaganmohan et al., 2021). The changes in CBF connectivity were explored in a variety of neurological diseases, such as Alzheimer’s disease (Zheng et al., 2019) and Parkinson’s disease (Shang et al., 2021). The majority of the differences between ASL and BOLD-FC networks were observed within the brain areas constituting the corresponding networks. In general, BOLD networks showed a stronger overall level of FC, with the exception of higher FC in several specific regions of CBF networks (Jann et al., 2015).

### Limitations

Several inevitable limitations must be acknowledged in this study. First, our sample size was moderate, which may limit the generalization of our results. Second, all subjects wore earplugs during the MRI scan, but the noise of the scanner is inevitable. This may affect the metabolism degree of attention network. Furthermore, the definition of acute and chronic tinnitus has not reached a uniform standard. Although the “Clinical Practice Guideline: Tinnitus” published in the United States in 2014 and the “A multidisciplinary European guideline for tinnitus: diagnostics, assessment, and treatment” published in 2019 both recommend that chronic tinnitus should last longer than 6 months, some studies used different standards such as 3 months (Besteher et al., 2019) and 4 years (Vanneste et al., 2011). Different classification criteria may have different results. Finally, we did not use longitudinal follow-up study in this study. If tinnitus patients were followed up regularly and grouped studies were conducted according to the outcome of the condition, the results might be more convincing. These limitations should be taken into consideration in further research.

## Conclusion

In summary, chronic tinnitus is different from acute tinnitus with increased CBF and CBF connectivity in several auditory and non-auditory brain regions. Multiple brain networks, including cognition, attention, emotion, and memory networks may be involved in the chronicity of tinnitus. Our study investigated the different brain neural mechanisms between acute and chronic tinnitus and emphasizes the potential use of ASL and CBF properties in the tinnitus field, which may bring us a better understanding of the neuropathological mechanisms underlying tinnitus chronicity.

## Data Availability Statement

The original contributions presented in the study are included in the article/supplementary material, further inquiries can be directed to the corresponding authors.

## Ethics Statement

The studies involving human participants were reviewed and approved by Research Ethics Committee of the Nanjing Medical University. The patients/participants provided their written informed consent to participate in this study.

## Author Contributions

JH and J-JX designed the experiment, analyzed the data, and drafted the manuscript for the work. SS and HC helped to acquire the clinical and fMRI data. XY helped to revise the manuscript critically for important intellectual content. JQ and YW did the financial support, review, and final approval of the manuscript to be published. All authors have read and approved the final manuscript.

## Conflict of Interest

The authors declare that the research was conducted in the absence of any commercial or financial relationships that could be construed as a potential conflict of interest.

## Publisher’s Note

All claims expressed in this article are solely those of the authors and do not necessarily represent those of their affiliated organizations, or those of the publisher, the editors and the reviewers. Any product that may be evaluated in this article, or claim that may be made by its manufacturer, is not guaranteed or endorsed by the publisher.
